# Novel Polyglutamine Model Uncouples Proteotoxicity from Aging

**DOI:** 10.1371/journal.pone.0096835

**Published:** 2014-05-09

**Authors:** Nakeirah T. M. Christie, Amy L. Lee, Hannah G. Fay, Amelia A. Gray, Elise A. Kikis

**Affiliations:** Biology Department, The University of the South, Sewanee, Tennessee, United States of America; National Institute for Medical Research, Medical Research Council, London, United Kingdom

## Abstract

Polyglutamine expansions in certain proteins are the genetic determinants for nine distinct progressive neurodegenerative disorders and resultant age-related dementia. In these cases, neurodegeneration is due to the aggregation propensity and resultant toxic properties of the polyglutamine-containing proteins. We are interested in elucidating the underlying mechanisms of toxicity of the protein ataxin-3, in which a polyglutamine expansion is the genetic determinant for Machado-Joseph Disease (MJD), also referred to as spinocerebellar ataxia 3 (SCA3). To this end, we have developed a novel model for ataxin-3 protein aggregation, by expressing a disease-related polyglutamine-containing fragment of ataxin-3 in the genetically tractable body wall muscle cells of the model system *C. elegans*. Here, we demonstrate that this ataxin-3 fragment aggregates in a polyQ length-dependent manner in *C. elegans* muscle cells and that this aggregation is associated with cellular dysfunction. However, surprisingly, this aggregation and resultant toxicity was not influenced by aging. This is in contrast to polyglutamine peptides alone whose aggregation/toxicity is highly dependent on age. Thus, the data presented here not only describe a new polyglutamine model, but also suggest that protein context likely influences the cellular interactions of the polyglutamine-containing protein and thereby modulates its toxic properties.

## Introduction

Polyglutamine (polyQ) expansions in certain proteins account for nine neurodegenerative disorders including Huntington's Disease (HD), Kennedy's Disease (KD), spinal and bulbar muscular atrophy (SBMA), dentatorubral pallidoluysian atrophy (DRPLA) and spinocerebellar ataxias (SCA) types 1, 2, 3, 6, 7 and 17. Each of these diseases is caused by an expansion of a polyQ-encoding CAG trinucleotide repeat, albeit in different genes coding for different proteins. The ataxin-3 protein consists of a globular domain followed by a natively unstructured, polyQ-containing, C-terminal tail [1]. While expanded polyQ leads to protein destabilization of some polyQ-containing proteins, recent evidence suggest that polyQ-expanded ataxin-3 is not significantly destabilized relative to the non-pathological forms of the protein [2], [3]. Nonetheless, ataxin-3 proteins harboring polyQ expansions aggregate under near-native conditions, and these aggregates are an important hallmark of disease [4].

The cytotoxicity of polyQ expansions has been demonstrated by expressing polyQ peptides in various tissues of the model organisms *Drosophila* [5] and *C. elegans* [6]–[8]. Specifically, a polyQ tract alone, outside the context of its normally diseases-associated protein, has been fused to YFP and expressed in various *C. elegans* tissues including body wall muscle cells [6], neurons [8] and intestinal cells [7]. An early model in which polyQ::YFP was expressed in *C. elegans* body wall muscle cells [6] was of the first, along with the *Drosophila* model [5], to demonstrate that polyQ alone has toxic properties, and that this toxicity is not restricted to neurons. In fact, the polyQ peptide fused to YFP has been shown to be toxic to *C. elegans* muscle cells in a polyQ length-, age-, and aggregation-dependent manner [6]. These findings were striking in the extent to which they recapitulated key aspects of polyQ diseases. Thus, these invertebrate models have become highly tractable tools for the study of polyQ protein dynamics and toxicity. Specifically, the polyQ::YFP model has been utilized to identify cellular factors that when knocked down by RNAi alter polyQ protein aggregation [9], [10]. These studies revealed molecular chaperones, and other components of the stress-response machinery to be genetic factors involved, positively or negatively, in the polyQ aggregation pathway [9], [10]. In addition to uncovering a role for key stress response proteins in polyQ aggregation, these findings revealed the power of *C. elegans* as a tool to elucidate the genetics of polyQ-mediated cytotoxicity.

Further pursuing the involvement of molecular chaperones in polyQ protein aggregation, it has been shown that polyQ physically associates with certain molecular chaperones, leading to the hypothesis that the sequestration of vital cellular factors by polyQ protein aggregates may underlie the mechanism of disease action [11], despite a fast on-off rate [12]. Additionally, it is has been proposed that the large-visible-aggregates associated with polyQ disorders are themselves inert, cytoprotective species that form via a highly toxic oligomeric intermediate [13], [14].

Despite the headway made toward understanding the underlying cellular dysfunction leading to polyQ-mediated toxicity, many questions remain unanswered, not the least of which is to consider the effect of the specific disease-associated proteins in which the polyQ tracts are normally found. The widespread expression of the nine disease-associated proteins and the fact that the resulting diseases present with unique symptoms, underscore the importance of considering the protein context of the polyQ expansion. Recent characterization of the structure of the polyQ-containing N-terminal domain of the HD-associated Huntingtin (Htt) protein has indicated that while the polyQ tract can adopt numerous secondary structures (alpha helix, random coil, or extended loop), its structure is influenced by the surrounding protein sequence [15].

We are specifically interested in the polyQ disease-associated protein ataxin-3 (AT3) in which an expansion of a polyglutamine-encoding CAG repeat is the genetic determinant for MJD. MJD, like the more well-studied Huntington's Disease (HD), is an age-dependent disorder in which polyQ-length correlates inversely with the age of disease onset. Most affected individuals begin to experience disease symptoms between 30-50 years of age. Symptoms of MJD include motor dysfunction and in its severest forms, difficulty eating and swallowing [16]. However, unlike HD, cognitive dysfunction is not associated with MJD. The age-dependence for MJD onset (and the onset of other protein folding disorders) is often thought to correlate with a general decline in protein folding homeostasis (proteostasis) that occurs during aging, such that eventually a cell becomes unable to fold or clear the polyQ-expanded protein.

The full-length AT3 protein contains a cysteine protease-like domain, a Josephin domain, and ubiquitin interacting motifs. AT3 has been shown to act, via its ubiquitin interacting motifs, in protein degradation pathways [17]. Despite the deubiquitinase activity associated with normal, native, AT3 protein, MJD is caused not so much by the loss of function of AT3, but instead by a dominant toxic-gain-of-function phenotype associated with the polyQ expansion.

To study the toxic properties of the polyQ-expanded AT3 protein in *C. elegans* neurons, this protein was previously expressed under control of the pan neuronal promoter F25B3.3 [18]. It was shown that AT3 aggregates in a polyQ length-dependent manner and that such aggregation was associated with motor defects [18]. Additionally, it was shown that both the full-length protein and a C-terminal cleavage product, thought to be especially toxic and relevant to MJD, form large-visible-aggregates in *C. elegans* neurons [18].

Here, we describe a new polyQ protein folding/aggregation model, in which the polyQ-containing C-terminal domain of the AT3 protein was expressed, with various polyQ lengths, in *C. elegans* body wall muscle cells. This model allows not only for the comparison of AT3 in different tissue types (muscle and neuronal), but also for the comparison of the toxic effects of polyQ alone and polyQ in the context of a disease-associated protein fragment within a tissue that is relatively large, easy to visualize, and readily amenable to gene knockdown by RNAi.

We demonstrate that the polyQ-containing C-terminal domain of AT3 forms aggregates in *C. elegans* body wall muscle cells in a polyQ length-dependent manner and that aggregation of this protein correlates with toxicity, as demonstrated by motor defects. However, unlike some previously described polyQ models [6], [19], aging is not a significant modifier of the aggregation and toxicity of this C-terminal fragment in body wall muscle cells. Furthermore, external proteotoxic stress, in the form of heat shock, does not affect aggregation of this AT3 fragment regardless of age.

## Materials and Methods

### Plasmid constructs

The P*_unc-54_*257cAT3(Q_n_)::YFP gene constructs were generated by inserting the AT3 C-terminal fragment (M)QGSSRNISQDMTQTSGTNLTSEELRKRREAYFEKQQQK(Qn)GDLSGQSSHPCERPATSSGALGSDLGDAMSEEDMLQAAVTMSLETVRNDLKTEGKK (Uniprot ID P54252, isoforms 2–5) from the previously published P*_F25B3.3_*257cAT3(Q_n_)::YFP plasmid [18] into the BamHI site of the pPD30.38Q0::YFP plasmid [6]. Plasmid constructs were obtained that had 45 or 63 glutamines, indicated as P*_unc-54_*257cAT3(Q45)::YFP or P*_unc-54_*257cAT3(Q63)::YFP, herein referred to as AT3CT(Q45) or AT3CT(Q63), respectively.

### *C. elegans* strains, crosses, and culture

*C. elegans* were cultured according the standard methods [20]. Specifically, animals were maintained at 20°C on NGM agar plates seeded with *E. coli* (OP50). The P*_unc-54_*YFP (herein referred to as YFP) line was previously published and expresses an integrated YFP transgene in body wall muscle cells [6]. For the generation of transgenic animals, 50 ng/µL of DNA encoding AT3CT(Q45) or AT3CT(Q63) were micoinjected into the gonads of adult N2 (wild type) hermaphrodites to generate multiple independent lines transmitting extrachromosomal arrays. For heat shock assays, ATCT3 lines were generated that co-express a fluorescent reporter for HSP70 (C12C8.1) gene expression (P*_C12C8.1_*mCherry) by crossing P*_C12C8.1_*mCherry males to YFP, AT3CT(Q45) or AT3CT(Q63) hermaphrodites. Homozygous lines, or, in the case of YFP, P*_C12C8.1_*mCherry, double homozygous lines were identified by single worm PCR using primers specific for YFP or mCherry.

### Fluorescence microscopy

For fluorescent images of transgenic *C. elegans,* live animals were immobilized with 2 mM levamisole and mounted on 2% agarose pads. For actin staining, animals were fixed with 4% paraformaldehyde. Actin filaments were stained with phalloidin (Molecular Probes/Life Technologies, Grand Island, NY) as previously described [21]. Images were obtained with a Ziess Axio Observer A1 (Oberkochen, Germany) inverted compound fluorescence microscope. For FRAP assays, live animals at day 1 of adulthood were treated with levamisole as above. FRAP was performed on a Zeiss LSM510 META (Oberkochen, Germany) confocal microscope under a 63× water objective as previously described [8].

### Motility assays

Motility was determined as a function of thrashing in liquid. Individual L4 larvae or animals at day 4 of adulthood were picked to a 10 µL drop of M9 on a microscope slide and were given a 30 s adjustment period before counting thrashing rate. Thrashes (defined as the head crossing the vertical midline of the body) were counted for 60 s. A minimal n-number of n = 30 was assayed for each genotype or time point indicated.

### Lifespan assays

Thirty N2, YFP, AT3CT(Q45) or AT3CT(Q63) age-matched animals were grown at 20°C and lifespan was monitored until all animals had died.

### Heat Shock assays

For heat shock treatment, 10–20 L4 larvae or animals at day 4 of adulthood were placed on NGM plates seeded with OP50. These plates were sealed with parafilm, placed in a plastic bag, and submerged in a 34°C water bath for 15 min as previously described [22]. Recovery after heat shock was for 6 hrs. This allowed time for the expression of the mCherry protein which served as a reporter for heat shock gene expression.

### Western blot analysis

100 fluorescent L4 larval stage animals were ground in liquid N_2_ and boiled in Laemmli sample buffer for 5 min prior to loading on a 10% polyacrylamide gel (SDS-PAGE). Following transfer to a PVDF filter, immunodetection was with RDye800 conjugated anti-GFP antibody (Rockland, Gilbertsville, PA). Visualization was with an Odyssey system from Li-Cor (Lincoln, NE).

### Native protein gels

Native lysates were obtained as previously described [23]. Protein was extracted from 100 fluorescent animals to mitigate the need for a loading control. Samples were run on 6% polyacrylamide gels without SDS. Visualization of in-gel YFP fluorescence was with a BioRad Gel Doc EZ imaging system, using UV or cyber green sample trays (BioRad Laboratories, Hercules, CA). Presumptive monomers, oligomers, and aggregates were defined by their relative migration rates.

## Results and Discussion

### Expression of a C-terminal cleavage fragment of human ataxin-3 in *C. elegans* body wall muscle cells causes polyQ length-dependent aggregation

Here, we describe the pathogenesis of a polyQ-containing fragment of AT3 in the body wall muscle cells of transgenic *C. elegans.* Wild type (N2) animals were transformed with gene constructs for the expression of a biologically relevant C-terminal cleavage product of AT3. Proteolytic cleavage of a number of neurodegenerative disease-associated proteins has been implicated in disease progression. With respect to MJD, evidence suggests that a proteolytic cleavage product, in which the N-terminal 257 amino acids have been removed, accumulates in the brains of MJD patients and is especially toxic in mammalian cell culture and in transgenic mice [24]–[27]. More recently, this fragment has been shown to be toxic and aggregation-prone in *C. elegans* neurons [18]. We are interested in whether different tissue types are differentially susceptible to the toxic effects of certain disease-associated polyQ-containing proteins. We come to this question from the perspective that different tissue types, each with their own proteome, differ with respect both to their proteostasis network (proteins that affect the synthesis, folding, and clearance of other proteins) [28] and with respect to their metastable protein load [29]–[32].

Thus, we asked whether this AT3 C-terminal fragment is likewise toxic to *C. elegans* body wall muscle cells. We expect that this will ultimately allow for the comparison of the aggregation/toxicity of this protein fragment to that of our previous described polyQ-alone model and to that of the AT3 C-terminal fragment in different tissue types.

**Figure 1:**
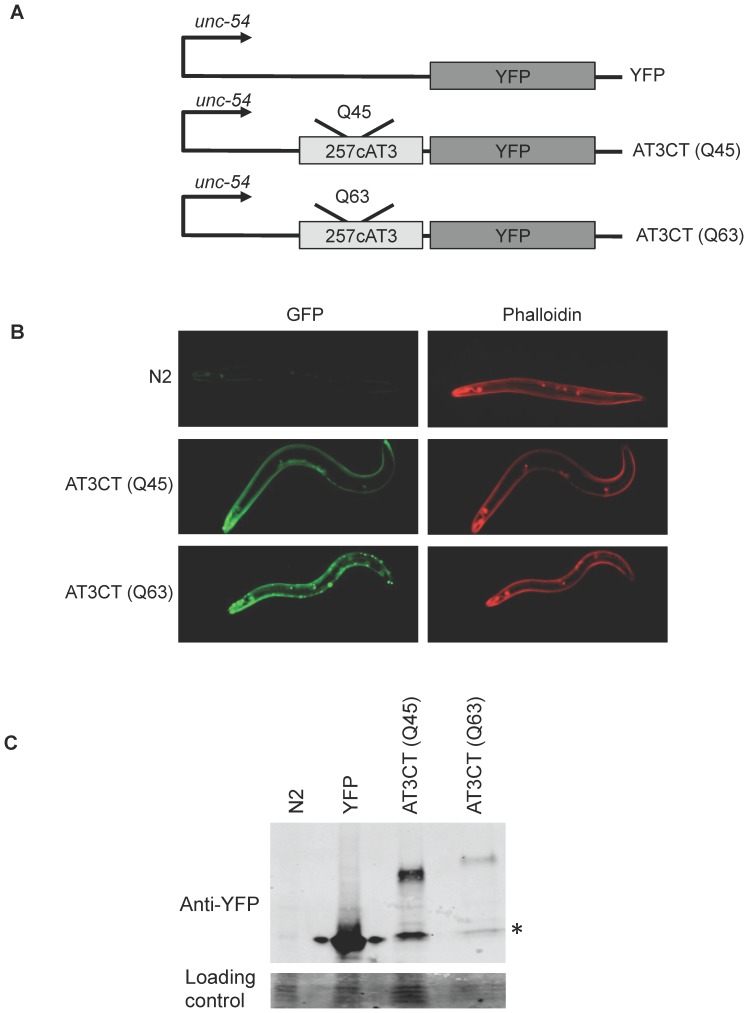
Expression of polyQ-expanded AT3CT in *C. elegans* body wall muscle cells. A. Schematic representations of gene constructs. The polyQ-containing C-terminal domain (lacking the N-terminal 257 amino acids) of AT3 was fused to YFP and expressed in body wall muscle cells under the control of the *unc-54* promoter to generate P*_unc-54_*257cAT3(Q_n_)::YFP, herein referred to as AT3CT(Q_n_) B. Fluorescent micrographs showing fixed N2 (wild type), AT3CT(Q45), and AT3CT(Q63) animals imaged for YFP fluorescence (green) or phalloidin-stained actin filaments (red). C. Western blot with an anti-GFP antibody showing relative protein levels for *C. elegans* expressing YFP alone or AT3CT(Q45) or AT3CT(Q63). The asterisk (*) represents protein running as a GFP monomer. In the AT3CT (Q45) and AT3CT (Q63) lanes, these bands seem to represent cleavage fragments that are likely artifacts of protein extraction.

To express the AT3 C-terminal cleavage fragment (257cAT3) in body wall muscle cells, we expressed it under the control of the myosin heavy chain promoter, *unc-54*. 257cAT3 was C-terminally fused to YFP for visualization, resulting in 257cAT3(Q_n_)::YFP, herein referred to as AT3CT(Q_n_) for the sake of simplicity. Because polyQ-length is known to affect the aggregation and toxicity of polyQ-containing disease-associated proteins [33]–[35], we generated two lines, one with an intermediate polyQ length that we predicted to be close to the threshold for aggregation (Q45), and a longer, likely pathogenic length (Q63) **(** **Fig. 1A** **)**.

Consistent with AT3CT(Q45) being close to the aggregation threshold for this protein in *C. elegans* body wall muscle cells, very few fluorescent foci were visible in young adult animals, with most of the protein resulting in diffuse fluorescence **(** **Fig. 1B** **)**. This is in contrast to AT3CT(Q63) young adults that mostly accumulated fluorescent foci, suggestive of protein aggregation **(** **Fig. 1B** **)**.

Because protein levels can have a significant impact on aggregation propensity and dynamics, we examined the relative protein levels in our transgenic lines by western blot analysis. Only lines accumulating similar levels of AT3CT(Q_n_) protein were compared in this study. Also, importantly, our AT3CT(Q_n_) lines express less protein than our YFP control **(** **Fig. 1C** **)**, allowing us to conclude that any toxic effects of the AT3CT(Q_n_) protein are due to intrinsic properties of that protein, and not due to excessively high expression levels.

**Figure 2:**
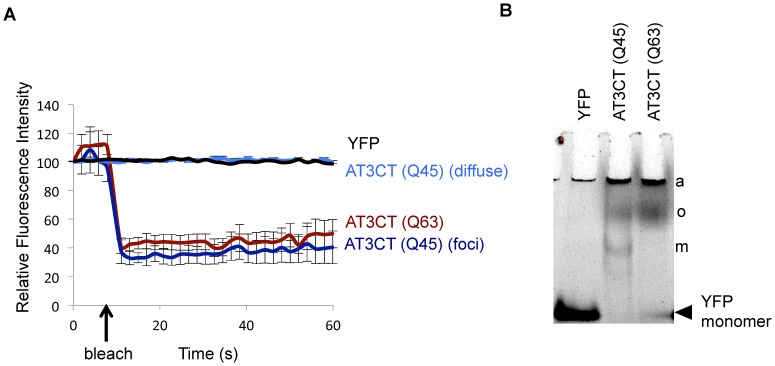
Aggregation of AT3CT in *C. elegans* body wall muscle cells. A. Fluorescence Recovery after Photobleaching (FRAP) of diffuse fluorescent protein in YFP or AT3CT(Q45)-expressing animals, or fluorescent foci in AT3CT(Q45) or AT3(Q63)-expressing animals. Time of bleach is indicated with an arrow. Fluorescence was monitored for 60 s. B. Native gel showing the YFP-containing protein species that accumulate in lines expressing YFP alone, AT3CT(Q45), or AT3CT(Q63). Presumptive aggregates (a), oligomers (o), and monomers (m) are indicated. Fluorescent protein species were visualized under UV light.

Because protein aggregation is an important hallmark of MJD and other neurodegenerative disorders [36], [37], we sought to determine whether the fluorescent foci observed in the AT3CT(Q63)-expressing animals, and, to a lesser extent, in AT3CT(Q45)-expressing animals, are true protein aggregates. To address this, we performed Fluorescence Recovery After Photobleaching (FRAP) of the large-visible-aggregates as well as areas of apparently diffuse fluorescence in AT3CT(Q45) and YFP alone. As shown in **Fig. 2A**, AT3CT(Q45) and AT3CT(Q63) foci fail to recover fluorescence within 60 s following photobleaching with a finely focused laser. This lack of fluorescence recovery is indicative of little to no protein mobility, and is consistent with the AT3CT(Q63) protein forming true, immobile, protein aggregates in *C. elegans* body wall muscle cells. Notably, AT3CT(Q45) protein forms very few foci in young adult animals. This differs from previously reported findings that polyQ-alone, fused to YFP, in *C. elegans* body wall muscle cells displays an aggregation threshold near Q35, with Q40 and longer resulting in a fully aggregated state [6]. These findings are consistent with aggregation propensity being modulated by the protein context in which the polyQ tract is embedded within the AT3CT fragment. We expect that this is likely due to differential interactions of AT3CT with members of the proteostasis network (specifically molecular chaperones or degradative pathways) compared to polyQ alone.

The few aggregates that did accumulate in day 1 adult AT3CT(Q45) animals were found in body wall muscle cells in the head. Most of the AT3CT(Q45) protein appeared as regions of diffuse fluorescence. FRAP analysis of these regions revealed recovery kinetics indistinguishable from that of YFP alone, indicating that this protein is highly mobile (not aggregated).

While FRAP assays are able to reveal whether certain foci are aggregates, they do not provide information regarding relative amounts of different protein species present in a given animal at a given time, and they do not provide information about protein species that are too small to detect using traditional confocal microscopy. This is especially significant, firstly because of the apparently low relative abundance of aggregated species in AT3CT(Q45) animals compared to AT3CT(Q63) and secondly, because recent evidence suggests that large-visible-aggregates may not be the toxic species. Instead, smaller oligomeric species, perhaps on an aggregation pathway, are the likely toxic species responsible for disease symptoms [38].

To examine all AT3CT protein species in a given population of animals at a given time, we separated extracted protein by size on native PAGE. Specifically, total protein was extracted from mixed populations (not age-matched) of *C. elegans* expressing either YFP as a control, AT3CT(Q45), or AT3CT(Q63) protein expressed in body wall muscle cells. Natively fluorescent YFP proteins were detected via in-gel YFP fluorescence. As shown in **Fig. 2B**, the AT3CT(Q45) protein accumulated as quickly migrating monomers, aggregates which were too large to enter the gel, and proteins of intermediate migration rate which likely represent oligomers; however, the possibility remains that intermediate-sized species represent monomers in complex with other proteins. For AT3CT(Q63), very little of the protein was detected as a monomer, with most of the protein forming large oligomers near the top of the gel or aggregates that were too large to enter the gel. Thus, these data, taken together with the data in **Fig. 1B**, indicate that the AT3CT protein aggregates in a polyQ length-dependent manner, and that polyQ length significantly modulates not only the amount of large-visible-aggregates, but also shifts the ratios of monomeric: oligomeric: aggregated species.

### Expression of a polyQ-containing fragment of human ataxin-3 in *C. elegans* body wall muscle cells results in polyQ length-dependent toxicity

**Figure 3:**
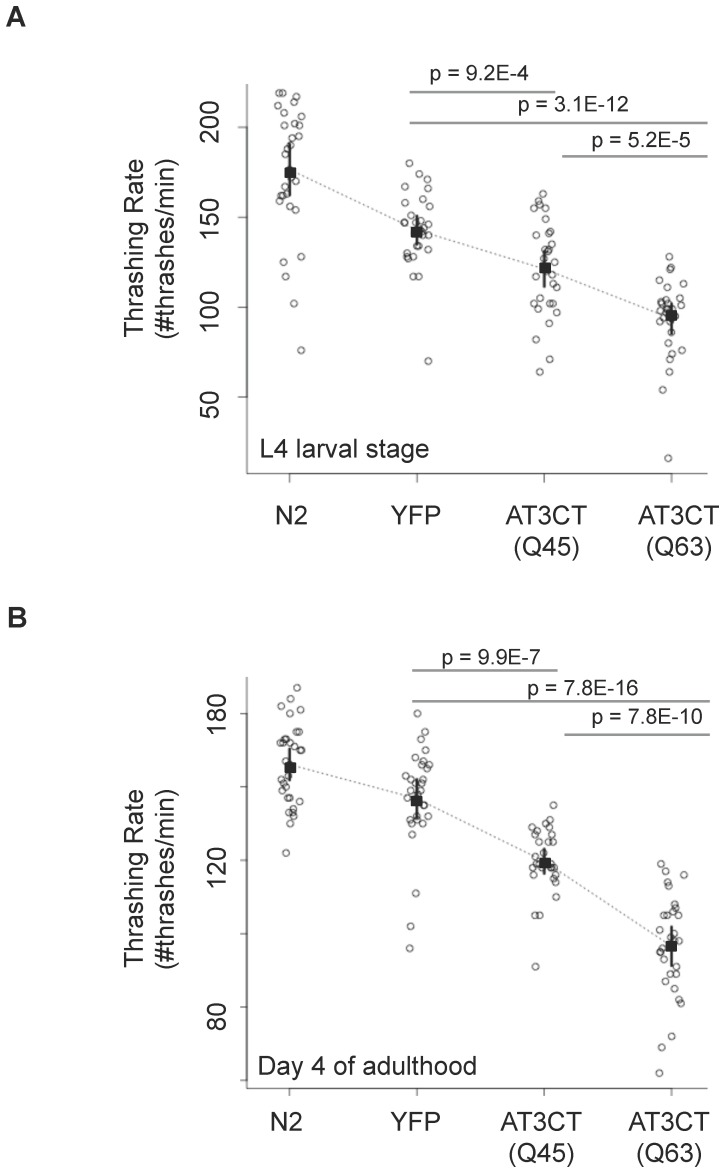
The toxic effects of AT3CT expression in *C. elegans* body wall muscle cells. Motility was determined as a measure of thrashing rate in liquid for L4 larvae (A) or animals at day 4 of adulthood (B). All 30 individual data points are represented on a dot blot. The symbol “▪” represents means. 95 percent confidence intervals are indicated. P-values are the results of pairwise student t-test.

The finding described above, namely that both AT3CT(Q45) and AT3CT(Q63) proteins formed a detectable amount of oligomeric species, and the fact that AT3 with an expanded polyQ tract is the determinant for MJD, led us to ask whether these proteins have toxic effects in *C. elegans* body wall muscle cells. To address this, we monitored the ability of N2 (wild type), YFP-expressing, or AT3CT(Q45 or Q63)-expressing L4 larval stage animals to thrash in liquid, a movement that is dependent on body wall muscle cell function. Consistent with previous findings in neurons [18], the expression of AT3CT protein in body wall muscle cells significantly reduced thrashing rate (**Fig. 3A**). Specifically, while the expression of YFP alone in body wall muscle cells impaired the ability of the animals to thrash in liquid as compared to wild type, an even more significant reduction in motor function was observed in animals expressing AT3CT(Q45 or Q63). The mean thrashing rate for YFP-expressing animals was 143 thrashes/min, whereas the mean for AT3CT(Q45) was 121 thrashes/min and 94 thrashes/min for AT3CT(Q63). Individual data points for each of 30 animals measured for each of the four genotypes (N2, YFP, AT3CT(Q45), and AT3CT(Q63)) are shown in **Fig. 3A** to indicate the full range of data.

To determine whether the observed differences in thrashing rate between genotypes were statistically significant, we performed student t-tests between selected pairs of data, as shown (**Fig. 3A**). The results indicate that the observed differences in thrashing rate between YFP-expressing animals and those expressing AT3CT(Q45) are statistically significant with a p-value of 8.9E-4. Additionally, the difference in thrashing rate between YFP-expressing animals and those expressing AT3CT(Q63) is also statistically significant with a p-value of 2E-12. Finally, the difference in thrashing rate between AT3CT(Q45)- and AT3CT (Q63)-expressing animals is likewise statistically significant with a p-value of 5E-5.

These data demonstrate a polyQ length-dependent toxicity, such that increasing polyQ length in the context of the AT3CT protein resulted in increased toxicity to body wall muscle cells. This is consistent with the toxicity observed for this same protein in *C. elegans* neurons [18], as well as previous findings for polyQ alone fused to YFP and expressed in *C. elegans* body wall muscle cells [6], and with findings in patient-derived neurons [39].

### Ataxin-3 C-terminal fragment aggregation and toxicity are not significantly modulated by aging

MJD and other polyQ disorders are diseases of aging, such that as an affected individual ages, the toxic effects of the mutant (polyQ-expanded) protein become more pronounced, while at the same time, more aggregated protein accumulates [40]–[43]. To address whether these phenomena are recapitulated in our AT3CT(Q_n_) muscle model, we first asked whether an increase in AT3CT(Q45 or Q63) toxicity is observed as *C. elegans* age. To that end, thrashing assays were performed at day 4 of adulthood (**Fig 3B**). While toxic effects of the AT3CT(Q45) and AT3CT(Q63) proteins were observed at day 4 of adulthood, the relative toxicity when compared to either N2 (wild type) or YFP-expressing animals was no more pronounced than in L4 larval stage animals. Specifically, the average thrashing rate of AT3CT(Q45) L4 larvae was 69% that of N2 at the L4 larval stage and 82% that of N2 at day 4 of adulthood. Likewise, the average thrashing rate of AT3CT(Q63) animals was 53% that of N2 at the L4 larval stage, and 66% that of N2 at day 4 of adulthood. We attribute the apparent decreased toxicity over time to a pronounced decrease in motility of older, wild type animals compared to L4 larvae.

**Figure 4:**
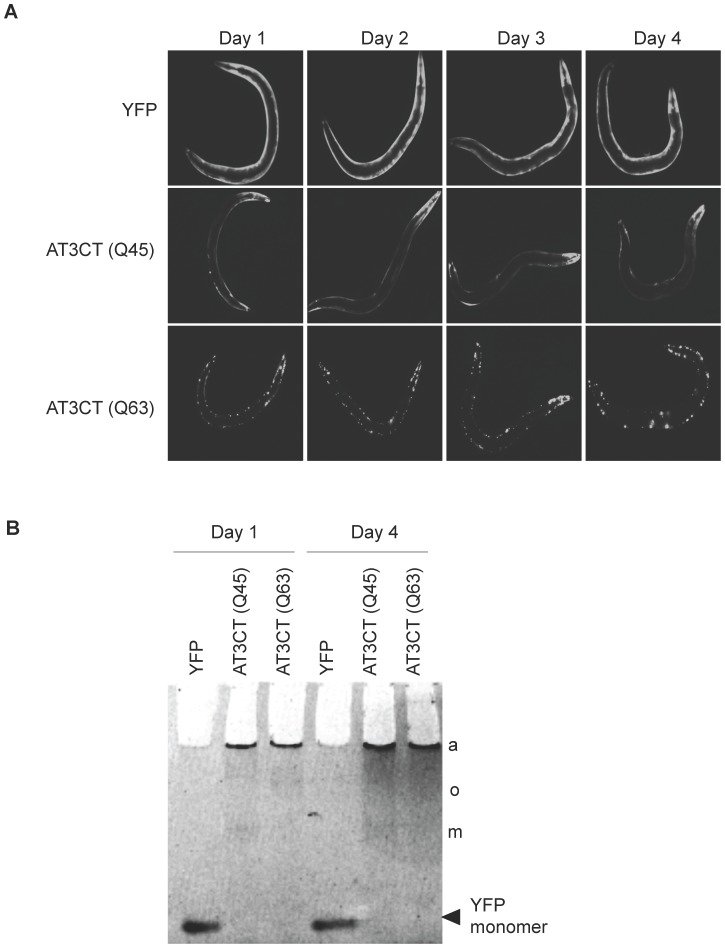
Effect of aging on AT3CT aggregation. A. Micrographs showing YFP fluorescence in YFP- or AT3CT(Q45 or Q63)-expressing animals. B. Native gels showing the relative distribution of presumptive monomers (m), oligomers (o), and aggregates (a) at day 1 as compared to day 4 of adulthood.

We further asked whether AT3CT(Q45 or Q63) aggregation becomes more pronounced during aging, and/or whether there is a marked increase in oligomer formation during aging. To address this, we first examined the pattern of AT3CT(Q45 or Q63) fluorescence in body wall muscle cells of animals at days 1–4 of adulthood. As shown in **Fig. 4A**, there was no marked increase in the accumulation of fluorescent foci in either AT3CT(Q45) or AT3CT(Q63). This is in contrast to the observed increase in aggregate formation in transgenic *C. elegans* expressing a polyQ35 tract alone, fused to YFP, in body wall muscle cells [6]. Additionally, it has been shown that while age-dependent aggregation of AT3CT(Q_n_) proteins was observed in some neurons, other neurons showed very little aggregation, or very little changes in aggregation during aging [18]. Thus, the apparent lack of age-dependent changes on AT3CT(Q_n_) aggregation in body wall muscle cells of *C. elegans* may suggest a cell-type specific effect on the aggregation propensity of these proteins. We further examined the protein content of these AT3CT(Q45 and Q63) animals by native gel analysis and found that even at day 4 of adulthood, there was still an observable amount of monomeric and oligomeric species present in AT3CT(Q45) and oligomeric AT3CT(Q63) (**Fig. 4B**). This is consistent with the lack of a pronounced increase in AT3CT(Q_n_) fluorescent foci in body wall muscle cells and indicative of little effect of aging on AT3CT(Q45 or Q63) aggregation.

We further tested whether the expression of the aggregation-prone, disease associated protein, AT3CT(Q45 or Q63), in body wall muscle cells affects lifespan. As shown in **Fig. S1**, there was no statistically significant difference in lifespan between wild type animals or those expressing YFP alone, in body wall muscle cells, and those expressing AT3CT(Q45 or Q63).

### *C. elegans* expressing ataxin-3 C-terminal fragment respond normally to temperature stress throughout aging

**Figure 5:**
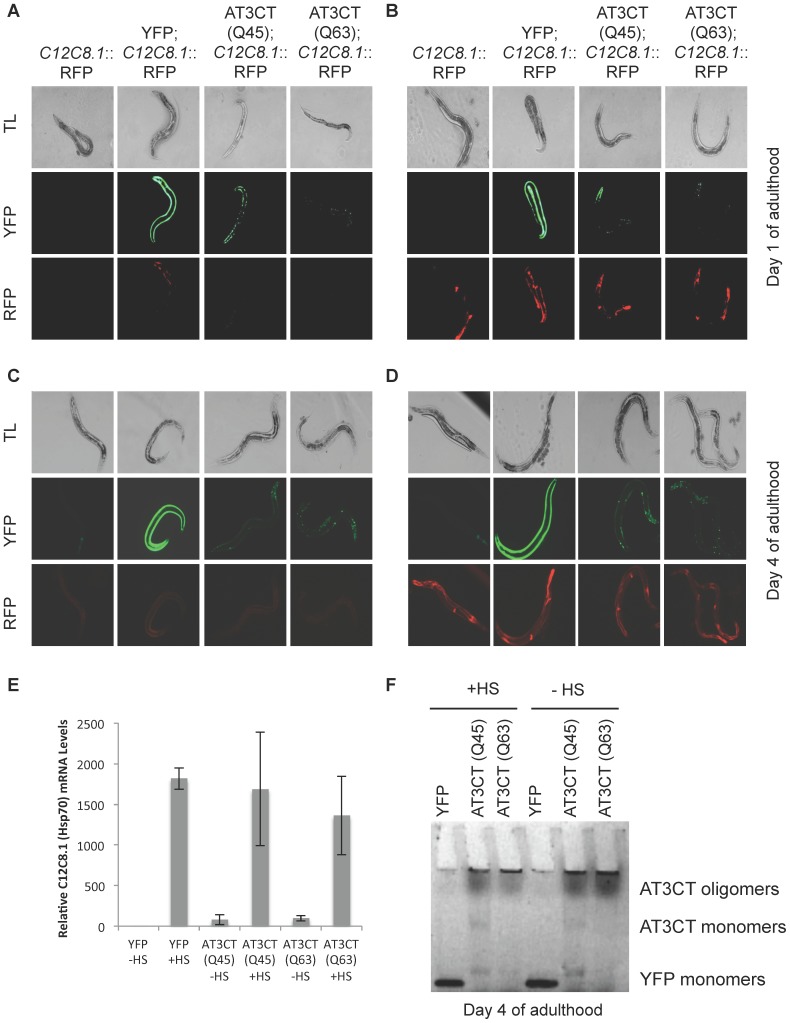
Effect of Heat Shock on *C. elegans* expressing AT3CT in body wall muscle cells. Animals expressing a reporter for the heat shock response (P_C12C8.1_mCherry) or co-expressing this reporter along with YFP, AT3CT(Q45), or AT3CT(Q63) were imaged at day 1 of adulthood before (A) or after (B) a 34°C/15 min heat shock, or at day 4 of adulthood before (C) or after (D) a 34°C/15 min heat shock. E) qRT-PCR showing the relative expression levels of the endogenous C12C8.1 mRNA before and after heat shock. F) Native gel showing in-gel fluorescence from samples taken from YFP, AT3CT(Q45), or AT3CT(Q63) animals after heat shock (+HS) or without heat shock (-HS) at day 1 of adulthood.

polyQ::YFP protein aggregation has been shown to be modulated by the activity of HSF-1 [6]. Nonetheless, the constitutive expression of misfolded and aggregation-prone polyQ::YFP has not been shown to constitutively induce a heat shock response (HSR), despite the fact that the HSR is generally accepted to be induced by the accumulation of heat-denatured proteins in the cytosol, at least in single cells in culture. To determine whether a polyQ expansion, in the context of a disease-associated protein fragment, results in the induction of a heat shock response in the absence of temperature stress, we crossed the AT3CT(Q45 and Q63) animals to lines harboring a reporter construct for C12C8.1 (heat-inducible cytosolic Hsp70) expression (P*_C12C8.1_*mCherry). As shown in **Fig. 5A**, there was no marked increase in reporter gene expression in day 1 adult AT3CT(Q45 or Q63)-expressing animals as compared to animals in which the reporter construct was present in an otherwise wild type (N2) background. This lack of observable stress induction continued to day 4 of adulthood, indicating that the cumulative effects of age and proteotoxic stress in the form of AT3CT(Q45 or Q63) protein expression are not sufficient to trigger heat shock gene induction (**Fig. 5C**). However, in light of recent evidence that expression of metastable proteins in *C. elegans* body wall muscle cells results in an upregulation of HSP90 gene expression in various tissues, and that HSP90 upregulation is associated with an inhibition of HSP70 gene expression [44], such a phenomenon underlying Hsp70 induction in AT3CT(Q_n_)-expressing animals cannot be ruled out.

We did ask whether the expression of AT3CT(Q45 or Q63) protein may affect the ability of these animals to launch a robust HSR under conditions of temperature stress. To test this, we exposed our double transgenic lines (expressing both the HSR reporter and the AT3CT protein) to a 34°C heat shock for 15 min. As shown in **Fig. 5B**, day 1 adult AT3CT(Q45 or Q63)-expressing animals were able to launch a heat shock response equally robust to that of wild type. This suggests that the accumulation of these disease-associated aggregation-prone proteins does not interfere with the heat shock response machinery.

We further asked whether the additional stress of aging might result in an impairment of the AT3CT(Q_n_) animals to launch a HSR. To address this, we examined C12C8.1 reporter gene expression at day 4 of adulthood in the AT3CT(Q45 and Q63) backgrounds. Even at day 4, these animals launched a HSR equally robust to that of wild type animals (**Fig. 5D**), indicative of aging, in conjunction with AT3CT(Q_n_) expression, playing little to no modulatory role in the induction of a cytosolic HSR. The organismal HSR was confirmed on the endogenous *Hsp70* genes, C12C8.1 via quantitative real-time RT-PCR. As shown in **Fig. 5E**, the AT3CT(Q45 or Q63)-expressing animals induced *Hsp70* gene expression to wild type levels upon temperature stress.

Temperature stress might be expected to increase the amount of misfolding/aggregation of aggregation-prone proteins, such as AT3CT(Q_n_). However, examination of the aggregation profiles of AT3CT(Q45 and Q63) protein before and after heat shock revealed no detectable shift from a less aggregated to a more aggregated state at day 1 (**Fig. 5F**), or at day four of adulthood (**Fig. S2**). This may be due to the normal induction of a presumably protective HSR.

In summary, these data indicate that expression of the AT3CT(Q_n_) protein is not sufficient to induce the canonical cytosolic stress response in the absence of heat shock at all ages examined. However, once exposed to temperature stress, these animals are fully able to induce heat shock gene expression, both in young (day 1) and older (day 4) animals. Nonetheless, this observable heat shock response does not result in apparent re-folding, or clearance, of aggregated or oligomeric AT3CT(Q_n_) protein.

## Conclusions

Taken together, the results presented here describe the polyQ length-dependent aggregation and toxicity of a novel model for AT3 aggregation in *C. elegans* body wall muscle cells. This model is set apart from other polyQ models in as much as no age-dependent aggregation was observed. This is in keeping, however, with findings for other disease-associated misfolded and aggregation-prone proteins, such as SOD1, which in its mutant forms is aggregated in body wall muscle cells from embryonic stages onward [23]. This suggests that the normal protein context in which the polyQ tract is embedded in a disease-associated protein significantly modulates the aggregation profile with respect to the relative amounts of monomeric, oligomeric, and aggregated protein, and thus likely affects the manner in which the polyQ protein associates with proteotoxic stress-related cellular pathways such as those involved in longevity.

## Supporting Information

Figure S1 Expression of AT3CT in body wall muscle cells does not affect *C. elegans* lifespan. Mean lifespan of animals expressing AT3CT(Q45 or Q63) in body wall muscle cells was compared to the mean lifespan of wild type or YFP-expressing animals. Each curve represents the mean lifespan of at least 40 animals at ∼20°C.
(TIFF)

Figure S2 Affect of HS on AT3CT protein aggregation at day 4 of adulthood. Native gel showing in-gel fluorescence from samples taken from YFP, AT3CT(Q45), or AT3CT(Q63) animals after heat shock (+HS) or without heat shock (-HS) at day 4 of adulthood.
(TIFF)
