# Supplementary figures and images for: Novel Polyglutamine Model Uncouples Proteotoxicity from Aging
Source: PLoS One. 2014 May 9;9(5):e96835. doi: 10.1371/journal.pone.0096835 (PMC4016013; doi:10.1371/journal.pone.0096835)

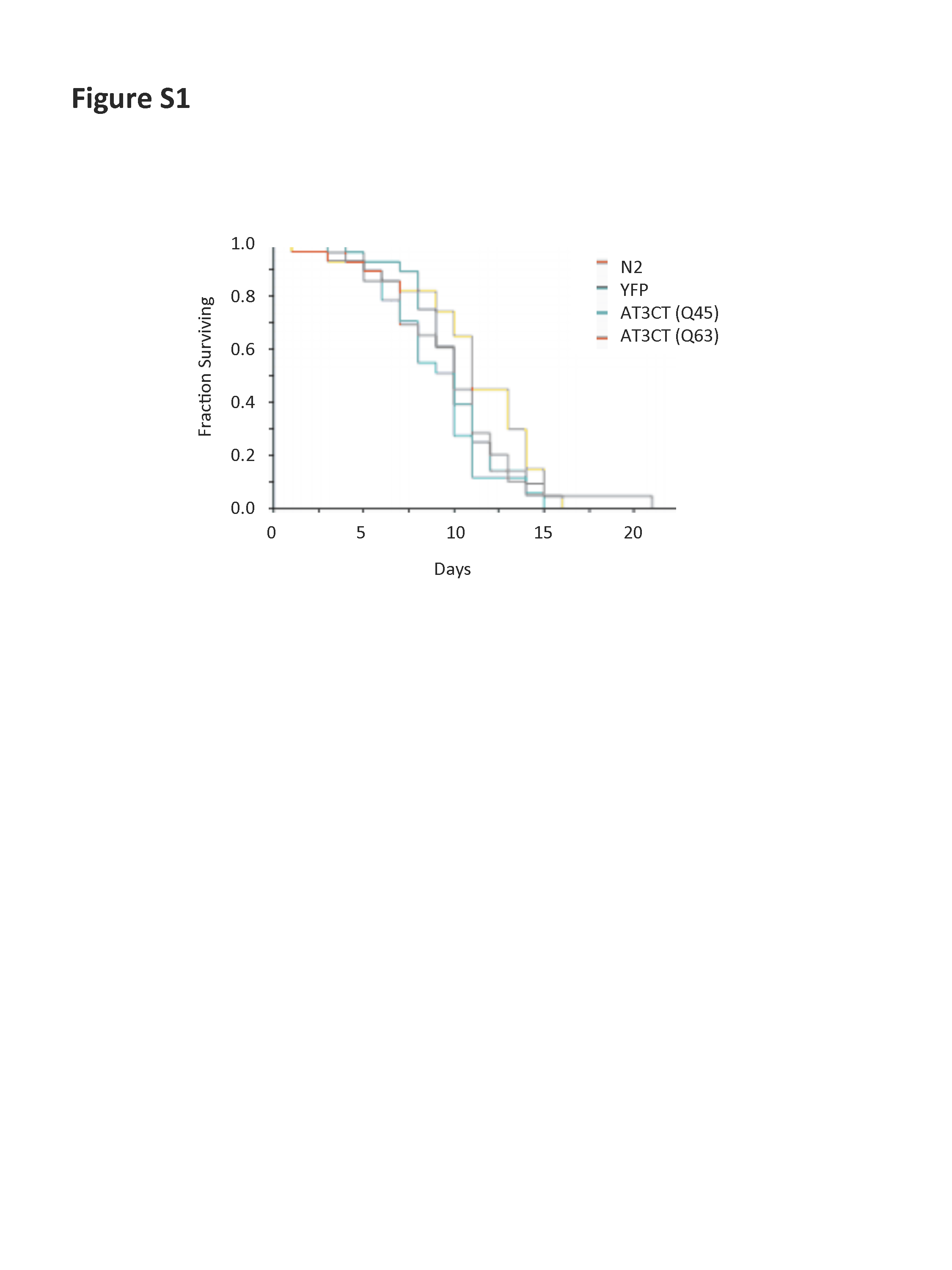

Supplement: Figure S1 — Expression of AT3CT in body wall muscle cells does not affect C. elegans lifespan. Mean lifespan of animals expressing AT3CT(Q45 or Q63) in body wall muscle cells was compared to the mean lifespan of wild type or YFP-expressing animals. Each curve represents the mean lifespan of at least 40 animals at ∼20°C. (TIFF) [file pone.0096835.s001.tiff]

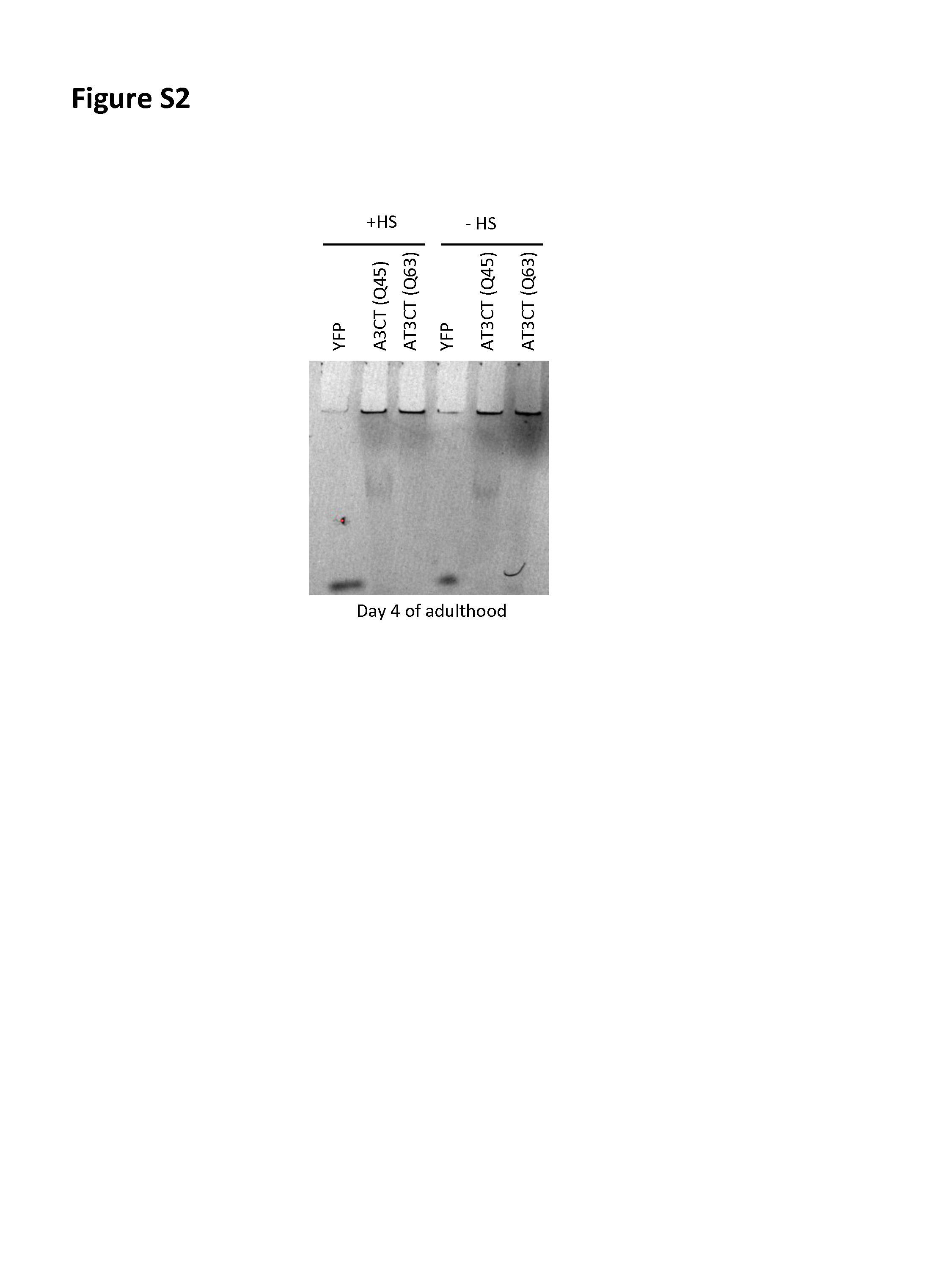

Supplement: Figure S2 — Affect of HS on AT3CT protein aggregation at day 4 of adulthood. Native gel showing in-gel fluorescence from samples taken from YFP, AT3CT(Q45), or AT3CT(Q63) animals after heat shock (+HS) or without heat shock (-HS) at day 4 of adulthood. (TIFF) [file pone.0096835.s002.tiff]
